# Incidence of age migration of colorectal cancer in younger population: Retrospective single centred-population based cohort study

**DOI:** 10.1016/j.amsu.2021.103214

**Published:** 2021-12-29

**Authors:** Safia Zahir Ahmed, Nicholas Cirocchi, Edward Saxton, Miss Katherine Brown

**Affiliations:** a Colorectal Department, Luton and Dunstable University Hospital NHS Foundation Trust, United Kingdom

**Keywords:** Age incidence rate, Colorectal cancer, Stage migration, Younger population

## Abstract

**Background:**

The positive correlation between the incidence of colorectal cancer and age is well known. However, current data demonstrates that there is noticeable age migration in the incidence of colorectal cancer towards young adults highlighting that this disease should no longer be considered to solely affect the elderly. The aim of the study is to analyse the changes in the trend for the age at which colorectal cancer presents, to identify if there is an appreciable age migration towards a younger population.

**Methods:**

This is a retrospective observational cohort study on a single-centred population. Data was obtained from The Luton and Dunstable University Hospital Colorectal Cancer Database. It was analysed from three separate decades from the years 1999, 2009, 2019 and 2020. All patients newly diagnosed with colorectal cancer in these time periods were included in this study. Gender, anatomical site, and the stage migration was also analysed.

**Results:**

A total of 598 patients were diagnosed with colorectal cancer in the specified time periods. The overall age-specific incidence rates had risen throughout the three decades. Notably the incidence rate had doubled between 1999 and 2009. In the younger adult population of <40 years, the incidence rate had risen seven-fold across three decades with a disproportionate increase among females compared to males. Stage migration is also analysed over the three decades with no evident difference in anatomical position of the tumour.

**Conclusion:**

There has been an upwards trend in the incidence of colorectal cancer among the younger population. This will have implications for investigation and screening in the future

## Highlights

• CRC is increasing in younger age population and is no longer an old age disease.
• There is an age migration in the incidence of CRC in the three decades studied.
• A change in the trend of initial stage presentation is noted in younger population.
• The incidence of rectal cancer is increased in patients younger than 60.
• Young patients with bleeding per rectum should be investigated for CRC.

## 1 Introduction

Colorectal cancer is the fourth most common cancer in the United Kingdom (UK) (accounting of 11% in 2017). There are reports of a rise in new cases of almost 100 everyday [1]. There is a strong positive correlation between incidence of colorectal cancer and an ageing population. Above 50 years of age, there is a sharp rise in the incidence of colorectal cancer. The cancer statistics report in 2017 found that 44% of new cases each year were aged 74 and above [2].

Until now, the emphasis has been on the diagnosis and prevention of colorectal cancer in older age groups via the two-week wait referral. The referral pathway is focuses on those aged over 60 with one or two symptoms. Patients aged below 60 are referred only if they have unexplained weight loss, rectal bleeding, or abdominal pain [3]. Concurrently, the bowel cancer screening programme is offered to those aged 60–74 [4]. It is projected that the proportion of the UK population aged over 85 is to increase two-fold by the year 2037, with an associated increase in the prevalence of the colorectal cancer [5].

According to the cancer registry data, the incidence rate of colorectal cancer in Europe during the last decade have remained stable [6]. However, when analysing the incidence rate in the younger age groups (25–49 years), there has been a significant increase of 41%. As a result of robust screening programmes, colorectal cancer incidence rates have decreased up to 6% in those aged between 60 and 75. It has been predicted that the colorectal cancer incidence rates will fall by 11% between 2014 and 2035 to 74 cases per 100,000 in 2035 [7].

Viuk et al. recently revealed that there has been an increase in incidence of colorectal cancer in young adults living in Europe, although there is some inconsistency among different countries. The paper claims that colon cancer incidence is rising compared to rectal cancers, however UK mortality data was unavailable [8]. Similar research papers have been published in the United States, Canada, Australia and New Zealand regarding the rise in the incidence of colorectal cancer in young adults [[9], [10], [11], [12]].

Studies have shown a variation in the distribution of colorectal cancer in young adults and have reviewed the gender proportion of tumours. Males are more prone to have rectal tumours, whereas females are more likely to have right-sided tumours. The anatomical subtype when linked with the age-specific incidence rate showed that North America is more prone to have rectal cancers whereas in Europe, the incidence is raised with colon cancer [13,14]. The young adult population within the UK was shown to have an increasing trend of both proximal and, most significantly, distal tumours. However, the recent review did not further specify if the distal tumours were left-sided or rectal cancer. The UK demographic data of the south-east of England, shows that the local population has an increase in the incidence of colorectal cancer when compared to the previous years(15).

The increase in incidence of colorectal cancer in young adults and the stage of presentation is very important to review and has not been reported on thus far. It will be interesting to investigate if there is a change in the stage of colorectal cancers at presentation or any stage migration in the younger population [15,16].

The aim of this study is to review the incidence rate of colorectal cancer in single basedpopulation; identify if there is any change in age migration, age-specific incidence rate, gender proportion, and the anatomical subtype; and to determine if there is any stage migration in the younger age group.

This will be the first study to categorise the stage at presentation in a younger cohort in a single based population and to analyse if there is any change to the specific-age incidence rate.

## 2 Methods

This is a retrospective observational cohort study conducted at Luton and Dunstable University Hospital. Ethical approval was not required. The study is registered with Research Registry, registration number research registry7336.

https://www.researchregistry.com/browsetheregistry#home/registrationdetails/618579a38f3316001e746609/[17]. The Study was conducted according to the principles of STROCSS criteria [18]. Data was retrieved from the Hospital Colorectal Cancer database and cross checked with the results data system. The data was selected from one year in three consecutive decades to analyse the changes in the trends of colorectal cancer. The years selected were 1999, 2009, 2019 and 2020. The latter was included to ascertain a reliable trend of the more recent incidence rate. All patients aged between 17 and 100 presenting with colorectal cancer were included. Patients with appendiceal adenocarcinoma, neuroendocrine tumour, melanoma, squamous cell carcinoma and lymphoma were excluded.

The following data was collected: patient demographics, the anatomical location of the cancer at presentation, and Tumour Nodes and Metastases (TNM) staging of the tumours at the initial presentation. This was obtained from Multi-Disciplinary Team (MDT) documentation and verified via imaging studies and considered as stage at presentation.

The data was divided into the following age groups: <40 years, 41–50, 51–60, 61–70, 71–80, 81–90, and 91–100. The patients in <40 age group were considered as young adults. The location of the tumour was categorised as: right (caecum-transverse colon), left (splenic flexure-sigmoid colon), rectum, and anal canal.

The TNM was recorded according to the NBOCAP staging and considered as: stage 1 (T1/T2,N0,M0), stage 2 (T3/T4,N0,M0), stage 3 (Any T, N1/N2,M0), Stage 4 (Any T, Any N, M1) [19].

The average population data was extracted from the Clinical Commissioning Group NHS Luton from the Office for National Statistics (ONS) with the area code of E38000102. A further breakdown of the population estimate was retrieved from the local authority (NOMIS office) based by single year of age and gender from the year 1998–2020 [20].

The age specific incidence rate was calculated by the newly diagnosed cases of colorectal cancer per year with the average population estimate per 100,000 person-year and the gender was stratified to the population estimate provided by the ONS [21].

The data was evaluated using SPSS version 28.0. The categorical and continuous variables were analysed using frequency, percentage and mean with standard deviation where appropriate.

## 3 Results

A total of 598 patients were identified with a new diagnosis of colorectal cancer in the years studied. 73 patients were diagnosed with colorectal cancer in 1999, 154 in 2009, 173 in 2019 and 198 in 2020.

In the age group of <40 years, the number of colorectal cancer cases had increased with 1 case in 1999, 3 in 2009, 7 cases in 2019 and 4 cases in 2020. The incidence rate in this age group had risen considerably in 2019 when compared to 1999, from 1.4 to 9.5 per 100,000 and 5.5 per 100,000 in 2020.

In the 41–50 age group, the incidence rate remained stable during the three decades but did show a rise in 2020 from 5 cases in 1999 to 14 cases in 2020 with an increase in the incidence rate from 22.5 to 52.1 per 100,000.

The 51–60 and 71–80 age groups demonstrated a gradual increase in the incidence rate shown in Fig. 1(a), Fig. 1(b). Conversely in the 61–70 years group, the incidence rate had decreased in 2019 when compared to 2009 data.

**Fig. 1(a):**
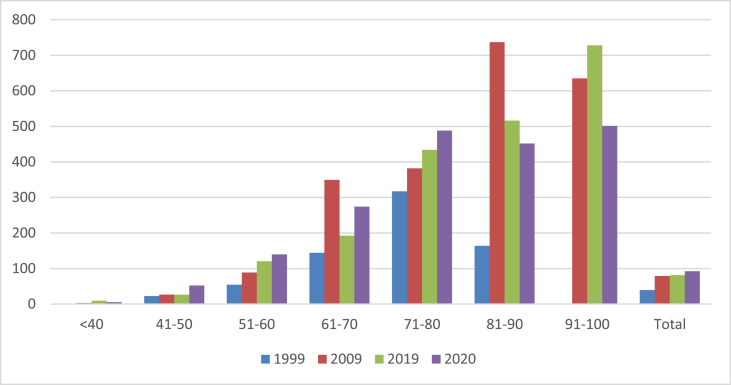
The Total Age incidence rate of patient who developed colorectal cancer (new cases per population x 100,000).

**Fig. 1(b):**
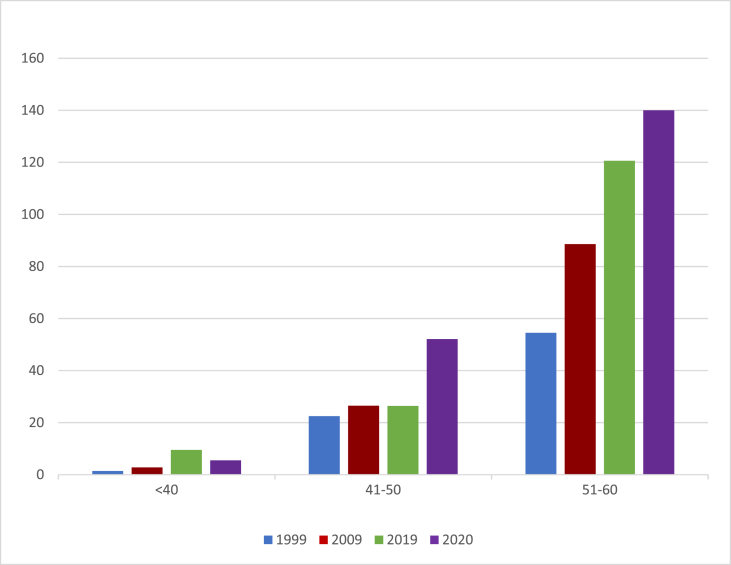
Age incidence rate of young age population <60 years.

The age-specific incidence rate with gender stratification shows no change in the trend regarding the male population in the <40 age group. The female population <40 age group on the contrary demonstrates a sharp rise from 1999 to 2020 with originally 0 per 100,000 to now 14.2 per 100,000.

In the female 41–50 group, between 1999 and 2020 there is a staggering four-fold increase in the incidence rate of colorectal cancer. Originally in 1999 with 18.3 per 100,000 rising to 78.2 per 100, 000.

**Fig. 2:**
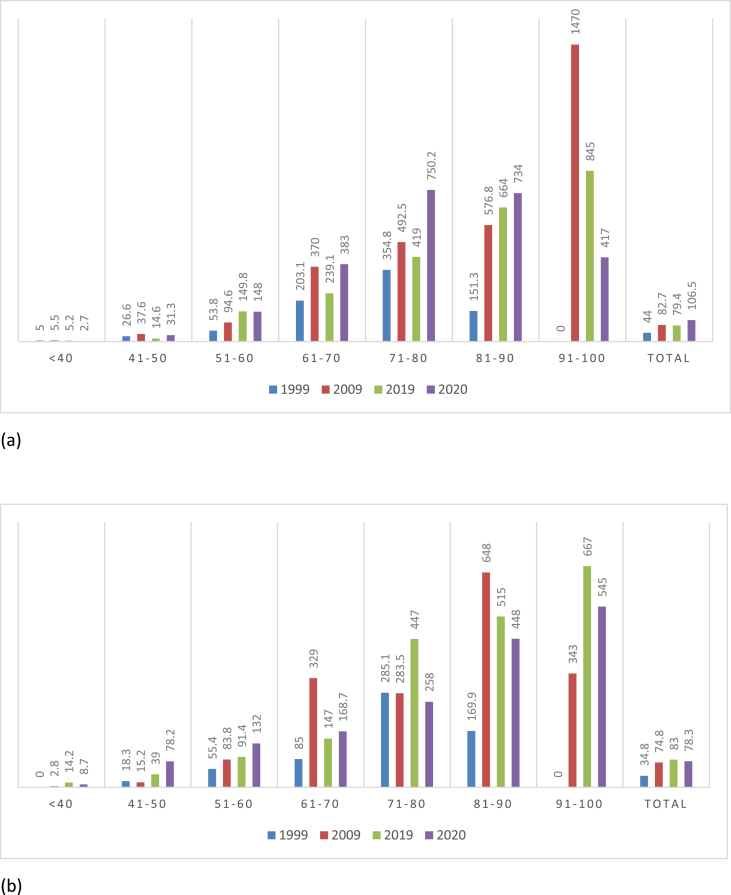
showing the age incidence of bowel cancer in male (a) and female population (b).

In the male 61–70 and 71–80 age groups, the occurrence of colorectal cancer has been steadily increasing, except for an isolated decrease in 2019. In comparison to, the female 61–70 and 71–80 age groups, there are no significant changes in the trend apart from isolated anomalies of an increase of incidence in 2019 (Fig. 2).

**Fig. 3:**
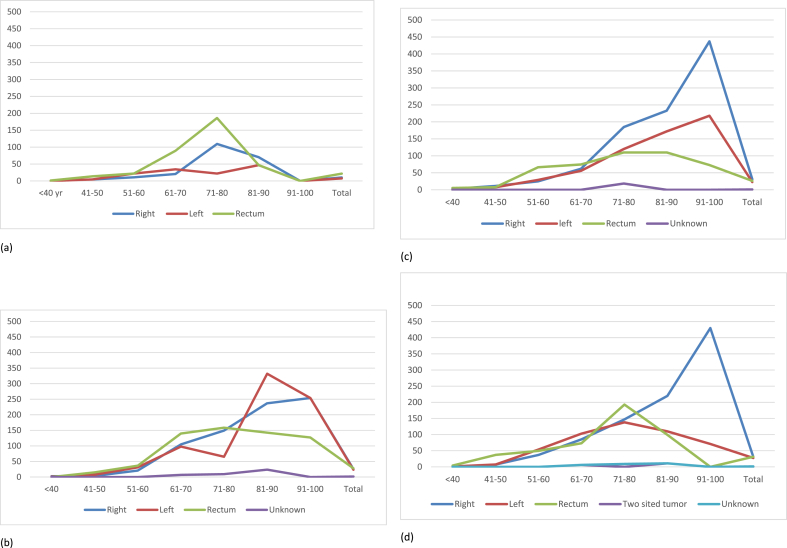
Age incidence stratified with anatomical site of bowel cancer in 1999(a), 2009(b), 2019(c) and 2020(d).

There was a noticeable change in trend of the incidence rate of right-sided and left-sided tumours in the <40 age group increasing in year 2009 and 2019. The trend of the rectal tumours raised noticeably from 1.4 per 100,000 in 1999, to 5.4 per 100,000 in 2019. This is a significantly higher incidence compared to other colorectal cancers. Similarly, in the 41–50 age group the same trend was noted, with a rise in the colonic tumours and an even larger rise in the incidence of rectal tumours. In 2019 the number of tumours occurring in the rectum was fewer when compared to 1999, 2009 and 2020. Between 2019 and 2020, there was a substantial rise in the incidence of rectal cancer was noted from 7.5 to 37.2 per 100,000 (Fig. 3).

Interestingly, there is a shift in the pattern of the tumours noted in the rest of the age groups. In 1999 above 50 years of age, there is a steep increase in the incidence of rectal tumours reaching a peak at the 71–80 age group followed by a fall in the incidence. However, when compared to 2009, in 2019 and 2020 there is a gradual increase in the incidence rate in the rest of the age group. Moreover, the numbers of left-sided and right-sided tumours have risen in 2009, 2019 and 2020 in the older age groups. Right-sided tumour numbers exceed the rest of the bowel cancers in later years.

**Fig. 4:**
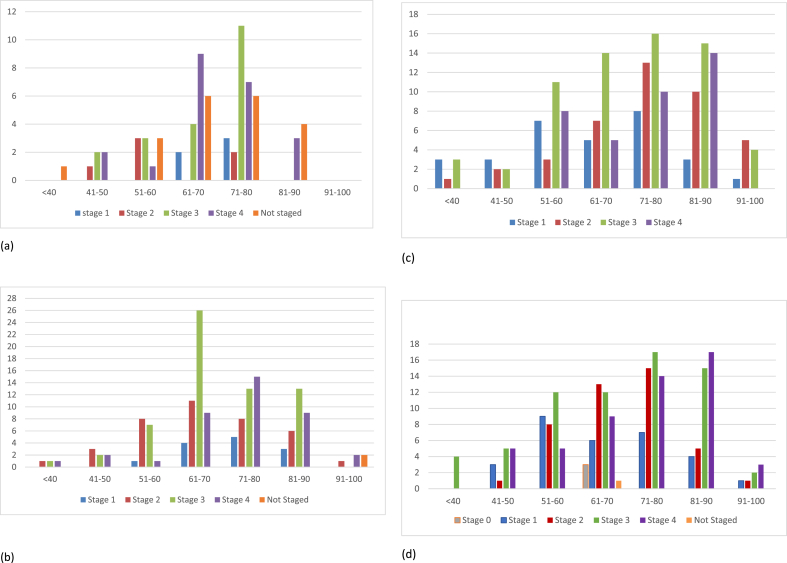
Age incidence stratified according to the TNM staging in 1999(a), 2009(b), 2019(c) and 2020(d).

The stage of the tumour could not be ascertained in the <40 age group in 1999. In 2009, the stage of the tumour showed an incidence rate of 1.3 per 100,000 at stage 2, 3 and 4 for the <40 age group. In 2019, there was an increase in incidence in stage 1 and 3, with further increase in the incidence of tumour presentation in stage 3 in 2020, increasing from 4.0 to 5.5 per 100,000 (Fig. 4).

In 41–50 age group, the majority of the tumours were stage 3 or 4 in 1999. In 2009 the majority of tumours presented as stage 2 and in 2019, most tumours presented as stage 1. In 2020, there was an increase in late presentation with the majority of the cases being diagnosed as stage 3 or 4.

In the 51–60 age group, the trend of the stage of tumour diagnosis were staged 2/3 in 1999 and 2009. The trend then migrates to the majority being stage 3/4 in 2019. In contrast, the 61–70 age group, the incidence of detection of the tumour migrated from stage 4 in 1999 to stage 2/3 in 2019 and 2020. Similar results we observed in the 71–80 age group.

## 4 Discussion

This retrospective observational review of this single hospital data base reveals an epidemiological shift in the increased incidence rate of the colorectal cancer in the younger population.

Colorectal cancer was previously thought to solely affect the elderly, however recent studies published in the US, Canada and Europe have revealed a dramatic change in the pattern of the raised incidence rate among young adults [22,23]. It was found that incidence rates of colorectal cancer in young adults had increased by two-fold when compared to the previous decades.

Based on these findings, it could be postulated that the risk factors to which people are exposed to within the environment have changed, causing an increased incidence of colorectal cancer in the younger population. Interestingly, Patel et al. found a rise in the colorectal cancer incidence of 6.7% per year in 15–29-year-olds and 2.4% per year in those aged 30–39. They associated this trend with the increase in prevalence to excess weight at a young age [24]. Similarly, Kantor et al. described the positive correlation between increased rates of adolescent and young adult obesity and incidence of the colorectal cancer( [25]. In England, the rate of obesity in children and adolescents has risen from 25% in 1995 to 40% in 2020 [26].

However, colorectal cancer in Europe was observed to rise from 2004(8). A recent study by Chambers et al. on the English female population (20–29 years) demonstrated a rising incidence rate since 1986. In the 30–39 age group, the colorectal cancer incidence rate began to rise from 1992 in females and from 2002 in males [15]. However, within this study no change was found to the trend of the colorectal cancer within the <40 and 41–50 male population groups. Conversely, a remarkable rise was found in the incidence of developing colorectal cancer at an early age within female population.

Interestingly, the rectal cancer incidence rate was higher in the <40 and 41–50 age groups compared to left and right-sided tumours. Left-sided tumours were also on the rise but was not as significant compared to rectal tumours. Right-sided tumours were more related to the older age groups.

The understanding of the risk factors that lead to developing colorectal cancer, including the anatomical site of tumour occurrence, is limited [27]. Obesity and reduced physical activity are thought to be related to colon cancer. Low fibre consumption and high-processed red meat intake is more associated with rectal cancer [28]. A recent review on the gut microbiome shows that the alteration of the structure and function of colon bacteria due to changes in lifestyle has a major role in development of colorectal cancer. The most common dysbiosis found on the sampling and biopsy were Fusobacterium, Porphyromonas, Bacteriodes and Prevotella [29]. These alterations to bacterial structure produce more hydrolytic enzymes and less bacterial enzymes in the distal colon. Pro-mutagenic and carcinogenic metabolites increase the DNA alkylation in the distal colon rather than in the proximal colon. The increased consumption of red meat and alcohol has shown to trigger pre-carcinogenic metabolites which increases the frequency of molecular polymorphisms in the rectum [30,31]. Furthermore, it is hypothesized that young adults are more likely to dine out at restaurants and fast-food establishments, leading to an increased risk of consuming carcinogenic foods [32]. A recent meta-analysis found that early-onset colorectal cancer is associated with: a history of cancer among close relatives increased alcohol consumption, hyperlipidaemia, obesity, decreased intake of dairy products and smoking. Other potential risk factors include: hypertension, the metabolic syndrome, ulcerative colitis and exposure to organ dusts.

Colorectal cancer in young adults often presents at an advanced stage and is aggressive in nature [13]. Teng et al. showed that 72% of young patients with colorectal cancer present with either metastatic or regional node involvement when compared to 50–58% of the general population [23]. This study is the first to analysis the stage migration in the younger population over three decades. There is no data available prior to 2012 in the cancer registry to review the stage of the presentation of the colorectal cancer as highlighted by recent study in England. This data showed similar results of age-specific staging of colorectal cancer presentation with regional and metastatic disease. A stage migration was found in the younger population. In addition, revealed that regional and metastatic disease in 1999 had improved to more localised disease in 2009 and 2019. In 2020, the frequency of presentation spiked with regional and metastatic disease, but this may be a delay of disease stage migration due to the COVID-19 pandemic. In age-specific staging for the 51–60 years group, there was a rise in the staging from localised to regional and metastatic disease. In the 61–70 and 71–80 age groups, there was a decreasing trend from metastatic to regional disease. This shows that the occurrence and presentation of the pathology could be due to robust screening within these age group.

These findings highlight that clinician should be aware of the increased incidence rate of colorectal cancer in young adults and that there should be a lower threshold for investigation of symptoms. Although the presentation frequency of colorectal cancer is fewer when compared older age groups, the rising trend is alarming. A revision to the colorectal cancer NICE guidelines should be considered due to these changing trends, which could avoid late presentation of colorectal cancer. Colorectal cancer awareness campaigns for young adults, such as “Never Too Young”, should be promoted. Faecal Immunochemical Tests (FIT) should be made available to the younger population, due to its 97% sensitivity at detecting colorectal cancer, to try and avoid late presentation [33]. Both primary and secondary care clinicians need to be aware that colorectal cancer is not only a disease that targets the elderly but can affect those under 60 years of age, where it can present at a more advanced stage and be more aggressive in nature.

The strength of this study is that it is from a single population in an area with a prospectively kept cancer data base, which gives high quality data specified to colorectal adenocarcinoma. The data was specified with the subsite of the tumour, stage presentation and the organ metastasis. One limitation of this study is that it is a single-centred analysis, therefore some discrepancy exists between the population extraction from the ONS and whether patients will present to the hospital. A minority of the patient population visiting from neighbouring areas could not be extracted exclusively. The results are also restricted with no continuous trend of years to review the exact figures, and no ethnicity of patients who developed colorectal cancer, which is another factor that should be considered.

In conclusion, this study shows that there is a change in the trends of colorectal cancer. The increased incidence rate in the younger population is alarming, as the presentation of the cancer is often at an advanced stage. The risk factors that are causing this change are still to be investigated to avoid the future burden of this disease on a younger population.

## Ethical approval

It is a retrospective observational cohort study and Ethical approval is not required.

## Funding

No Fund to Disclose.

## Author contribution

Ms Safia Zahir Ahmed- Study Design, data collection, Analysis, interpretation, writing of the manuscript and proof reading, Mr Nicholas Cirocchi- Data collection and Proof reading of the manuscript, Dr Edward Saxton- Correction of manuscript and proof reading, Miss Katherine Brown- Study Concept, Study Design and Proof reading.

## Registration of research studies

Name of the registry: Research Registry.

Unique Identifying number or registration ID: researchregistry7336.

Hyperlink to your specific registration (must be publicly accessible and will be checked):

https://www.researchregistry.com/browse-theregistry#home/registrationdetails/618579a38f3316001e746609/

## Guarantor

Miss Katherine Brown.

## Disclaimer

None to declare.

## Consent

Not Applicable.

## Provenance and peer review

Not commissioned, externally peer-reviewed.

## Declaration of competing interest

There is no conflict of interest.
